# Editor and Reader

**Published:** 1899-01

**Authors:** 


### Editor and Reader.

When the subscriber sends his money for a periodical he should be given his money's worth. Does he get it in the vast majority of medical journals? Do their editors take their calling seriously, even in the commercial sense of *quid pro quo?* If an editor does his work with professional ideals and aims he will try to meet and satisfy the needs of his subscribers. If he does it for the supposed honor of the position, he should certainly be no less conscientious. If he assumes the office for the salary he secures, he should at least earn the salary by attention to the demands of those who pay the expense. Hence, all motives unite in commanding editorial obedience as an officer in the service of his subscribers. Take up many journals and judge them by these standards. The original articles cost the editor nothing except the labor required to prepare them for the type setter. (How often is this done in a workmanlike manner?) Are the news of the profession secured by special correspondents, money being spent in the getting; or is the news not usually copyings from other journals, incomplete, and not seldom stale as the joke of a comic newspaper? We know of journals publishing no word of a report of the best medical societies meeting in their own city, or not doing so for several months after the dates of the meetings.

And then the abstracting of other journals—in what a nondescript, haphazard, scrappy way it has been done by one and all of us in the past! A subscriber could never be at all certain that a hundred desirable articles had not been missed even in the best of his journals; and he was certain that not one-tenth of the articles in the principal journals he did not take had been abstracted for him. Was this taking one's office seriously? Would it pass muster as workmanship in any lay review or newspaper? If a subscriber should rely on such reports (consisting of one, two, or three only, in each of the great departments, "under the charge of,") to keep him posted as to the work of his colleagues in all parts of the world, would he not find himself scientifically stranded at the end of the volume?

Lastly, as regards editorials, have our journals usually in the past summarized the trend of professional opinion and work, judged dispassionately, denounced inethicality with frankness, and tried to stimulate professional unity and progress?

To write thorough and systematic abstracts of the best literature requires a degree of scholarship and a conscientiousness of which few indeed have anything approaching an adequate conception. We have no desire unduly to "magnify our office," but we wish the profession to recognize the enormous labor done by the staff of our journal in a sincere atempt to present an epitome of the world's professional literature at once after its publication. So far as we know, no journal has ever undertaken such a job before. We are by no means satisfied with the present degree of perfection with which we are carrying out the scheme, although the unanimous voice of the profession in praise would justify contentment. We claim, however, that it is only by a still more generous professional support that we can bring about a complete realization of our aims, which is immediately to mirror the world's medical advances, in such a way as to be of the greatest service to our subscribers.

It is sometimes answered, "Oh, we are taking all the journals we can possibly read already." But is that an answer to our offer? We do not ask you to discontinue any that are truly professional in their management; but, candidly, do the majority of the others give you value received as we do? We frankly ask you to institute a comparison, even of a strict commercial kind, as to the relative number of pages, editorial labor, practicality, subscription-price, etc., and then answer the straightforward question, which one is giving the most for the money? For the time we may purposely leave out of the count literary and scientific values, and put the matter down on the bargaining basis. We are at present giving you at the rate of almost 3,000 double- column pages of matter for $3.00, or 10 pages for one cent! If cheapness is desirable where else can the bargainer find a similar offer? This may seem somewhat egotistic and as if basing the affair upon rather a low standard, but when the strictly professional journal must compete in the commercial market for place, it needs occasional mention that the commercial journals may be beaten on their own ground. If the physician uses the excuse that he already takes more journals than he can read, he may be answered by the query that even if he prefers supporting the lay-owned sort why should he prefer to pay a very much higher price for them? —*Philadelphia Medical Journal.*

We give our contemporary the benefit of the above free advertisement, because he expresses our own views so well.

